# Elevated red cell distribution width predicts poor outcome in young patients with community acquired pneumonia

**DOI:** 10.1186/cc10355

**Published:** 2011-08-11

**Authors:** Eyal Braun, Erel Domany, Yael Kenig, Yoav Mazor, Badira F Makhoul, Zaher S Azzam

**Affiliations:** 1 Department of Internal Medicine B, Rambam Health Care Campus, 1 Ha'aliya St. P.O.B. 9602 Bat Galim, Haifa 31096, Israel; 2 Rappaport Faculty of Medicine, Technion-Israel Institute of Technology, Efron St. P.O.B. 9649 Bat Galim, Haifa 31096, Israel; 3 Rappaport Family Institute for Research in the Medical Sciences, Technion, Efron St. P.O.B. 9649 Bat Galim, Haifa 31096, Israel

**Keywords:** Pneumonia, Red Blood Cell Width, Mortality, Prognosis, Complicated Hospitalization

## Abstract

**Introduction:**

Community acquired pneumonia (CAP) is a major cause of morbidity and mortality. While there is much data about risk factors for severe outcome in the general population, there is less focus on younger group of patients. Therefore, we aimed to detect simple prognostic factors for severe morbidity and mortality in young patients with CAP.

**Methods:**

Patients of 60 years old or younger, who were diagnosed with CAP (defined as pneumonia identified 48 hours or less from hospitalization) between March 1, 2005 and December 31, 2008 were retrospectively analyzed for risk factors for complicated hospitalization and 90-day mortality.

**Results:**

The cohort included 637 patients. 90-day mortality rate was 6.6% and the median length of stay was 5 days. In univariate analysis, male patients and those with co-morbid conditions tended to have complicated disease. In multivariate analysis, variables associated with complicated hospitalization included post chest radiation state, prior neurologic damage, blood urea nitrogen (BUN) > 10.7 mmol/L and red cell distribution width (RDW) > 14.5%; whereas, variables associated with an increased risk of 90-day mortality included age ≥ 51 years, prior neurologic damage, immunosuppression, and the combination of abnormal white blood cells (WBC) and elevated RDW. Complicated hospitalization and mortality rate were significantly higher among patients with increased RDW regardless of the white blood cell count. Elevated RDW was associated with a significant increase in complicated hospitalization and 90-day mortality rates irrespective to hemoglobin levels.

**Conclusions:**

In young patients with CAP, elevated RDW levels are associated with significantly higher rates of mortality and severe morbidity. RDW as a prognostic marker was unrelated with hemoglobin levels.

**Trial registration:**

ClinicalTrials.Gov NCT00845312

## Introduction

Community acquired pneumonia (CAP) is a major cause of severe morbidity and mortality. It is the sixth most common cause of death in the USA, and it is estimated that four million cases of CAP occur annually [1]. There is a worldwide increase in the number of hospitalizations due to CAP in the general population [2-4].

Much research has been conducted in recent decades to determine prognostic factors for adverse outcome in patients hospitalized for CAP, including concomitant diseases and laboratory parameters on admission [5]. Several prognostic scores were developed based upon these characteristics, such as the Pneumonia Patient Outcomes Research Team score [6]. Ghanem-Zoubi et al reported recently in a study that included 43% of patients with pneumonia that simple clinical score and mortality in emergency department sepsis scores were the most appropriate clinical scores to predict the mortality of patients with sepsis in general internal medicine departments [7]. Although there is a large body of evidence in this field in the general population, less focus was put in younger group of patients, even though several recent studies showed that there is an increasing number of hospital admissions due to CAP among patients less than 60 years old [8]. Recently, it was reported that procalcitonin is associated with the severity of illness in patients with severe pneumonia and appears to be a prognostic marker of morbidity and mortality [9].

Red Blood Cell Distribution Width (RDW) is a laboratory index used in the differential diagnosis of microcytic anemia. Recently, several studies showed that a high RDW index predicts severe morbidity and mortality in various cardiac conditions, such as acute and chronic congestive heart failure [10,11], pulmonary hypertension, [12] and stroke. [13]

Therefore, our primary aim was to determine prognostic factors that are associated with complicated hospitalization and 90-day mortality. The major secondary endpoint was to assess whether RDW is associated with adverse outcome irrespective of hemoglobin and white blood cell (WBC) levels.

## Materials and methods

Patients 60 years old or younger, who were diagnosed with CAP (defined as pneumonia identified 48 hours or less from hospitalization) between 1 March, 2005 and 31 December, 2008 were retrospectively analyzed for risk factors for severe morbidity or mortality. Data were collected from the Prometheus, Rambam Integrated Computer System for handling patients' medical records, and the 90-day mortality data were retrieved from the database of our hospital and the ministry of health.

Complicated hospitalization was defined as at least one of the following parameters: hospitalization longer than 10 days, admission to ICU and in- hospital mortality. Otherwise, the hospitalization was defined as uncomplicated. The Rambam Hospital Institutional Review Board approved the study. The need for informed consent was waived.

Exclusion criteria included transfer from another hospital, hospitalization for any cause other than CAP during the 30 days prior to admission, hospital-acquired pneumonia (defined as pneumonia which was diagnosed more than 48 hours after admission) or partial antibiotic treatment before admission.

The following data were retrieved from the electronic medical records of the patients:

(1) Malignancies: solid tumors, hematologic malignancies. (2) Pulmonary diseases: bronchial asthma, chronic obstructive lung disease, interstitial lung disease, bronchiectasis, permanent tracheostomy, lung malignancy, past history of thoracic radiotherapy, previous episode of pneumonia, and previous or current active smoker. (3) Immune suppression conditions: current chronic corticosteroid treatment, current or recent chemotherapy treatment, carrier of HIV, primary immune deficiency, history of bone marrow transplantation. (4) Cardiovascular diseases. (5) Chronic kidney disease. (6) Diabetes mellitus. (7) Liver cirrhosis. (8) Prior neurologic damage. (9) Chronic alcohol use. (10) Intravenous drug abuse. (11) Nursing house residents.

### Laboratory variables on admission

Serum glucose, creatinine, sodium, hemoglobin, WBC, RDW and blood urea nitrogen (BUN) were measured on admission.

Hemoglobin levels, mean corpuscular volume and RDW were measured on admission and prior to hospital discharge, using the Advia 120 Hematology Analyzer (Siemens Healthcare Diagnostics Deerfield, Illinois, USA). Glucose, BUN and creatinine levels were measured using the "Dimension" (Siemens Healthcare Diagnostics Deerfield, Illinois, USA).

RDW is reported as coefficient of variation (in percent) of red blood cell volume. The normal range for RDW in our laboratory is 11.5 to 14.5%. The correctness of this normal range was confirmed by analyzing RDW data in 17,293 ambulatory subjects who attended the Rambam Center for Preventive Medicine for a medical examination and health counseling. In this group, mean RDW was 13.1% (median 13.0%) with 95% confidence interval (CI) of RDW of 12.0 to 14.4%.

### Statistical analysis

Binary logistic regression analysis was used for the calculation of the odds ratios (OR) with 95% CI and *P *values in univariate analysis to identify association between patient characteristic and 90-day mortality and complicated hospitalization. Multivariate forward stepwise logistic regression was performed to assess the relation between patient characteristics: co-morbidities, laboratory results, and 90-day mortality or complicated hospitalizations.

Variables were selected as candidates for the multivariate analysis on the basis of the level of significance of the univariate association with 90-day mortality and complicated hospitalization (*P *< 0.1). Notably, there was no predilection in choosing RDW or any other variable in the statistical model.

The area under curve (AUC) was used as a measure of model of discrimination. The calibration of the prediction equation was assessed by comparing the observed and expected numbers of 90-day mortality or complicated hospitalization. The calibration of the prediction equation was assessed by comparing the observed and expected numbers of 90-day mortality or complicated hospitalization rate by decile of predicted risk. The Hosmer-Lemeshow goodness-of-fit statistic was calculated.

Comparing of patients characteristics from two groups (complicated and uncomplicated) was done by using chi-square test. Student's t-test was used to compare age between the two groups, whereas, length of stay was compared using Mann-Whitney nonparametric test.

Two-tailed *P *values of 0.05 or less were considered as statistically significant. All statistical analyses were performed using SPSS (Statistics Products Solutions Services; Armonk, New York, USA) 17.0 software for Windows; Redmond, Washington, USA.

## Results

The cohort included 637 patients; 63% were males, median age was 46 years, the in-hospital mortality rate was 6.6% and the median length of stay was five days.

### Univariate analysis of complicated hospitalizations and 90-day mortality

As shown in Table 1, 171 patients (26%) experienced complicated hospitalization; male patients and those with co-morbid conditions tended to have a complicated disease. Notably, patients who had concomitant diabetes mellitus, chronic liver diseases, interstitial lung diseases and past chemotherapy did not have a more complicated course of CAP (*P *= not significant).

**Table 1:** Baseline characteristics of the cohort with univariate analysis of risk factors for detection of complicated hospitalization

Characteristic		N_T _(%)	N_UC _(%)	N_C _(%)	Odds ratio (95% CI)	*P *value
		637	466 (73.2)	171 (26.8)		
Gender	Male	403 (63.3)	282 (60.5)	121 (70.8)	1.58 (1.082-2.305)	0.018
	Female	234 (36.7)	184 (39.5)	50 (29.2)	Reference	1.000
Age (years)	≤30	126 (19.8)	106 (27)	20 (11.7)	Reference	
	31-40	136 (21.4)	97 (29.2)	39 (22.8)	2.13 (1.163-3.904)	0.014
	41-50	144 (22.6)	109 (30.9)	35 (20.5)	1.70 (0.924-3.135)	0.088
	51-60	231 (36.3)	154 (49.6)	77 (45.0)	2.65 (1.528-4.596)	0.001
Co-morbid conditions						
Malignancy		60 (9.4%)	32 (6.9)	28 (16.4)	2.66 (1.546-4.563)	<0.0001
Prior neurologic damage		125 (19.6%)	57 (12.2)	68 (39.8)	4.74 (3.134-7.160)	<0.0001
Chronic renal failure		26 (4.1%)	15 (3.2)	11 (6.4)	2.07 (0.930-4.594)	0.075
Heart disease		56 (8.8%)	34 (7.3)	22 (12.9)	1.88 (1.063-3.310)	0.030
Lung disease*		193 (30.3%)	128 (27.5)	65 (38)	1.61 (1.115-2.337)	0.011
Asthma		41 (6.4%)	35 (7.5)	6 (3.5)	0.45 (0.185-1.084)	0.075
Chronic obstructive lung disease		77 (12.1%)	50 (10.7)	27 (15.8)	1.56 (0.941-2.585)	0.084
Lung malignancy		37 (5.8%)	20 (4.3)	17 (9.9)	2.46 (1.257-4.820)	0.009
Post chest radiation		31 (4.9%)	14 (3)	17 (9.9)	2.96 (1.074-8.146)	0.036
Immune deficiency		156 (24.5%)	98 )21)	58 (33.9)	1.93 (1.309-2.839)	0.001
Hematologic malignancy		45 (7.1%)	30 (6.4)	15 (8.8)	2.38 (0.944-5.982)	0.066
Corticosteroid therapy		76 (11.9%)	44 (9.4)	32 (18.7)	2.21 (1.347-3.619)	0.002
Nursing institution		52 (8.2%)	21 (4.5)	31 (18.1)	4.69 (2.612-8.427)	<0.0001
SBP on admission (mmHg)		137.3 ± 32.1	137.9 ± 36.1	136.6 ± 29.0	1.009 (0.942-1.082)	NS
DBP on admission (mmHg)		77.4 ± 16.1	77.8 ± 18.3	76.9 ± 12.6	1.011(0.945-1.081)	NS
Heart rate on admission (min^-1)^		86.7 ± 20.2	86.8 ± 20.6	86.7 ± 20.1	0.998 (0.926-1.077)	NS
Laboratory parameters						
BUN > 10.7 mmol/L		65 (10.2%)	35 (7.5)	30 (17.5)	2.62 (1.552-4.422)	<0.0001
Creatinine >114 μmol/L		88 (13.8%)	54 (11.6)	34 (19.9)	1.89 (1.183-3.031)	0.008
Hemoglobin <110 g/L		113 (17.7%)	68 (14.6)	45 (26.3)	2.11 (1.375-3.229)	0.001
Hematocrit <30 mg/dl		56 (8.8%)	31 (6.7)	25 (14.6)	2.42 (1.383-4.233)	0.002
Glucose >13.8 mmol/L		39 (6.1%)	24 (5.2)	15 (8.8%)	1.77 (0.903-3.454)	0.096
WBC <4 or >12 × 10^9^/L		347 (54.5%)	238 (51.1)	109 (63.7)	1.68 (1.174-2.416)	0.005
RDW >14.5%		218 (34.2%)	123 (26.4)	95 (55.6)	3.49 (2.419-5.023)	<0.0001

*P *< 0.05 represents significant statistical difference between patients with complicated and uncomplicated hospitalization

BUN, blood urea nitrogen; CI, confidence interval; DBP, diastolic blood pressure; N_C_, number of complicated admissions; ns, not significant; N_T_, all the patients that were included in the cohort; N_UC_, number of uncomplicated admissions as defined; RDW, red cell width distribution; SBP, systolic blood pressure; WBC, white blood cells.

* Includes bronchial asthma, chronic obstructive lung disease, interstitial lung disease, bronchiectasis, permanent tracheostomy, lung malignancy, past history of thoracic radiotherapy, previous episode of pneumonia, and previous or current active smoking.

As depicted in Table 1, patients who had disturbed renal function tests, anemia, abnormal WBC and elevated RDW on admission had a complicated hospitalization.

The median length of stay was 4 and 12 days in uncomplicated and complicated patients, respectively. In patients who had a complicated course of pneumonia, 90-day mortality was 31% as compared with 3.9% in uncomplicated patients (*P *< 0.03). Notably, the combination of elevated RDW and abnormal WBC count had the highest OR for complicated hospitalization and 90-day mortality (Tables 2 and 3 respectively). We found a statistically significant association between each element of the complicated hospitalization and elevated RDW. For length of stay of more than 10 days, linear regression showed B = 3.5 (95% CI = 1.8-5.1, *P *< 0.0001). For ICU admission logistic regression showed OR of 1.97 (95% CI = 1.2-3.1, *P *= 0.004), and for in- hospital mortality, binary regression showed OR of 3.5 (95% CI = 2.4-5).

**Table 2:** The combination of elevated RDW and abnormal WBC count had the highest odds ratio for complicated hospitalization

		n (%)	Complicated hospitalization (%)	Odds ratio (95% CI)	*P *value
		637			
RDW ≤14.5	4≤ WBC ≤12 × 10^9^/L	197 (30.9)	15.7%	Reference	
	WBC <4 or >12 × 10^9^/L	222 (34.9)	20.3%	1.36 (0.82-2.25)	0.23
RDW >14.	4≤ WBC ≤12 × 10^9^/L	93 (14.6)	33.3%	2.68 (1.5-4.77)	0.001
	WBC <4 or >12 × 10^9^/L	125 (19.6	51.2%	5.62 (3.34-9.45)	<0.0001

CI, confidence interval; OR, odds ratio; RDW, red cell width distribution; WBC, white blood cells.

**Table 3:** The combination of elevated RDW and abnormal WBC count disclosed the highest OR for 90-day mortality

		n (%)	90-day mortality (%)	Odds ratio (95% CI)	*P *value
		637			
RDW ≤14.5	4≤ WBC ≤12 × 10^9^/L	197 (30.9)	2.0%	Reference	
	WBC <4 or >12 × 10^9^/L	222 (34.9)	5.0%	2.5 (0.8-8.0)	0.119
RDW >14.	4≤ WBC ≤12 × 10^9^/L	93 (14.6)	8.6%	4.5 (1.3-15.5)	0.016
	WBC <4 or >12 × 10^9^/L	125 (19.6	15.2%	8.6 (2.9-26.1)	<0.0001

CI, confidence interval; OR, odds ratio; RDW, red cell width distribution; WBC, white blood cells.

### Multivariate analysis of complicated hospitalizations

All variables that were associated (*P *< 0.1) with complicated hospitalization were included in the initial multivariate prediction rule. Results of multivariate analysis are presented in Table 4.

**Table 4:** Results of multivariate analysis of risk factors for complicated hospitalization

		Without RDW					With RDW					RDW and abnormal WBC				
	**% from Total**	**Coef.**	***P *value**	**Adjusted odds ratio**	**95% confidence interval**		**Coef.**	***P *value**	**Adjusted odds ratio**	**95% confidence interval**		**Coef.**	***P *value**	**Adjusted odds ratio**	**95% confidence interval**	
					**Lower**	**Upper**				**Lower**	**Upper**				**Lower**	**Upper**

Prior neurologic damage	19.6	1.7	<0.0001	5.3	3.4	8.2	1.5	<0.0001	4.5	2.9	7.0	1.6	<0.0001	4.7	3.0	7.3
Heart disease	8.8	0.6	0.044	1.9	1.0	3.5										
Corticosteroid therapy	11.9	0.7	0.01	2	1.2	3.5										
Post chest radiation	4.9	1.4	<0.001	4.1	1.9	8.9	1.1	0.006	3.1	1.4	6.9	1.1	0.007	3.0	1.4	6.8
BUN >10.7 mmol/L	10.2	0.8	0.008	2.2	1.2	3.8	0.8	0.004	2.3	1.3	4.1	0.8	0.004	2.3	1.3	4.1
WBC <4 or >12 × 10^9^/L	54.5	0.6	0.004	1.8	1.2	2.7										
RDW ≤14.5%	34.2						1.0	<0.0001	2.9	1.9	4.2					
RDW ≤14.5% and WBC = normal	31												<0.0001	Reference		
RDW >14.5% and WBC = normal	49											0.5	0.048	1.7	1.0	3.0
RDW >14.5% and WBC = abnormal	20											1.4	<0.0001	4.1	2.6	6.5
Constant		-2.084					-1.96					-1.97				
Hosmer and Lemeshow Test			0.543					0.344					0.565			
Area under curve				0.7376	0.691	0.781			0.743	0.699	0.788			0.755	0.711	0.799

Elevated RDW levels on admission either alone or in combination of abnormal levels of white blood are associated with significant higher rates complicated hospitalization.

BUN, blood urea nitrogen; Coef, coefficient; RDW, red cell width distribution; WBC, white blood cells.

In a model that does not include RDW, variables that were associated with complicated hospitalization included prior neurologic damage, heard disease, prior corticosteroid treatment; post chest radiation state, BUN above 10.7 mmol/L and abnormal WBC.

Whenever RDW was included in the model, the variables that were associated with complicated hospitalization included prior neurologic damage, post chest radiation, BUN above 10.7 mmol/L and RDW above 14.5%. However, with the inclusion of both elevated RDW and abnormal WBC, the variables that were linked with complicated hospitalization included prior neurologic damage; post chest radiation BUN above 10.7 mmol/L and combination of elevated RDW and abnormal WBC. A model with RDW improved AUC_ROC _as compared with a model without RDW from 0.736 (95% CI = 0.691-0.781) to 0.743 (95% CI = 0.699-0.788); conceivably, the improvement was prominent in a model with the combination of RDW and abnormal WBC from 0.736 to 0.755 (95% CI = 0.711-0.799). The Hosmer-Lemeshev goodness-of-fit statistic across decile of risk was not statistically significant indicating little departure a perfect fit in all three models.

### Multivariate analysis of 90-day mortality

All variables that were found to be associated (*P*<0.1) with 90-day mortality in the univariate analysis (Table 5); were included in the initial multivariate prediction rule. As depicted in Table 6; in the first model that excluded RDW, variables that were associated with an increased risk of 90-day mortality included age 51 years or older, prior neurologic damage and immunosupression. Including RDW in the model demonstrated that the same variables in addition to elevated RDW were associated with increased 90-day mortality. Notably, the model that joined both elevated RDW and abnormal WBC; it was proved that this combination is significantly associated with increased mortality in addition to the three previous variables. The model with RDW improved AUC_ROC _as compared with a model without RDW from 0.807 (95% CI = 0.746-0.868) to 0.822 (95% CI = 0.766-0.879); while the model of combined elevated RDW and abnormal WBC improved AUC_ROC _from 0.807 to 0.828 (95% CI = 0.772-0.885). The Hosmer-Lemeshev goodness-of-fit statistic across decile of risk was not statistically significant, indicating little departure from a perfect fit in all three models.

**Table 5:** Univariate analysis of risk factors for detection of 90-day mortality

Characteristic		N_T_	90-day mortality n (%)	Odds ratio (95% CI)	*P *value
		637			
Gender	Male	403	28 (6.9)	1.17 (0.605-2.276)	0.64
	Female	234	14 (6.0)	Reference	1.000
Age groups (years)	≤30	126	5 (4.0)	Reference	
	31-40	136	4 (2.9)	0.73 (0.192-2.794)	0.65
	41-50	144	6 (4.2)	1.05 (0.313-3.543)	0.93
	51-60	231	27 (11.7)	3.2 (1.202-8.537)	0.02
Malignancy		60	11 (18.3)	3.95 (1.873-8.349)	<0.0001
Prior neurologic damage		125	22 (17.6)	5.25 (2.766-9.982)	<0.0001
Post chest radiation		31	17 (9.9)	3.56 (1.716-7.400)	0.001
Immune deficiency		156	19 (12.2%)	2.76 (1.461-5.221)	0.002
Corticosteroid therapy		76	10 (13.2%)	2.50 (1.178-5.328)	0.017
Nursing institution		52	10 (19.2%)	4.11 (1.893-8.942)	<0.0001
BUN >10.7 mmol/L		65	9 (13.8%)	2.62 (1.195-5.765)	0.016
Hemoglobin <110 g/L		113	12 (10.6%)	2.02 (0.999-4.100)	0.050
WBC <4 or >12 × 10^9^/L		347	30 (8.6%)	2.19 (1.101-4.365)	0.025
RDW >14.5%		218	27 (12.4%)	3.81 (1.979-7.324)	<0.0001

BUN, blood urea nitrogen; CI, confidence interval; N_T_, all the patients that were included in the cohort; RDW, red cell width distribution; WBC, white blood cells.

**Table 6:** Results of multivariate analysis of risk factors for 90-day mortality

		Without RDW					With RDW					RDW and abnormal WBC				
	**% from total**	**Coef.**	***P *value**	**Adjusted odds ratio**	**95% confidence interval**		**Coef.**	***P *value**	**Adjusted odds ratio**	**95% confidence interval**		**Coef.**	***P *value**	**Adjusted odds ratio**	**95% confidence interval**	
					**Lower**	**Upper**				**Lower**	**Upper**				**Lower**	**Upper**

Age ≥51 years	36.3	1.2	0.001	3.3	1.7	6.5	1.1	0.001	3.1	1.6	6.2	1.0	0.003	2.8	1.4	5.6
Prior neurologic damage	19.6	1.7	<0.0001	5.3	2.7	10.2	1.6	<0.0001	4.9	2.5	9.7	1.6	<0.0001	5.0	2.6	10.0
Immunedeficiency	24.5	1.1	0.001	3.1	1.6	6.2	0.8	0.001	3.1	1.6	6.2	0.9	0.012	2.5	1.2	5.0
RDW ≤14.5%	34.2						0.8									
RDW ≤14.5% and WBC = normal	30.9												0.016	Reference		
RDW >14.5% or WBC = normal	49.5											0.9	0.128	2.4	0.8	7.3
RDW >14.5% and WBC = abnormal	19.6											1.6	0.007	4.8	1.5	15.3
Constant		-4.187					-4.453					-4.937				
Hosmer and Lemeshow Test			0.310					0.363					0.708			
Area under curve				0.807	0.746	0868			0.822	0.766	0879			0.828	0.772	0885

Elevated RDW either alone or in combination with abnormal WBC, proved to be significantly associated with increased mortality.

Coef, coefficient; RDW, red cell width distribution; WBC, white blood cells.

### The association of RDW and complicated hospitalization

Ninety five (55.6%) patients with complicated hospitalization had increased RDW as compared with 123 (26%) patients with uncomplicated admissions (*P*<0.0001).

**Figure 1:**
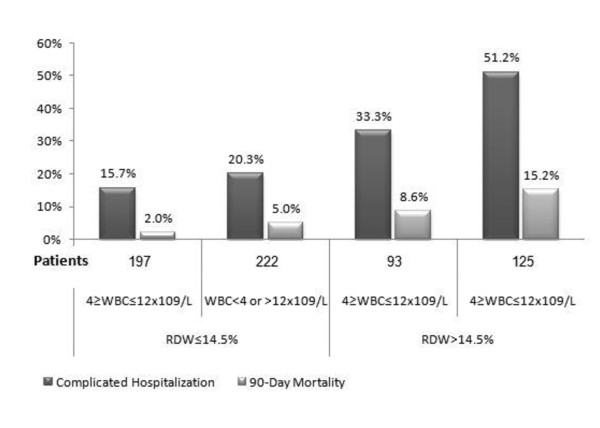
**The association between the mortality rate and complicated with RDW and the different white blood cell groups**. RDW, red cell width distribution; WBC, white blood cells.

Complicated hospitalization and mortality rates were significantly higher among patients with increased RDW regardless of the WBC count (Figure 1); the complicated hospitalization rate was 15.7% in patients with normal RDW and WBC and 20.3% in patients with only abnormal WBC count, as compared with 33.3% in patients with elevated RDW alone (*P *= 0.001) and 51.2% in patients with combined abnormal leukocyte count and increased RDW (*P*<0.001). As shown in Figure 1, the 90-day mortality was higher in patients with elevated RDW regardless of the WBC count; however, once again the combination of abnormal WBC and elevated RDW was significantly associated with the highest mortality rates.

**Figure 2:**
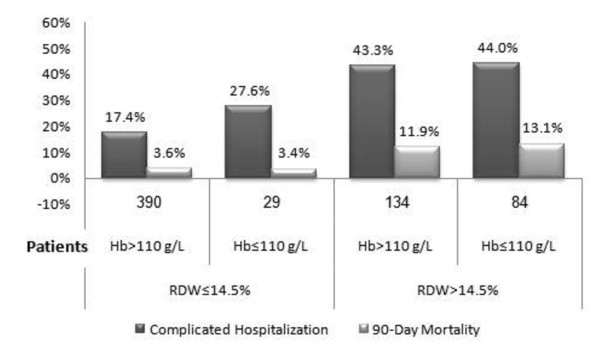
**The association between the 90-day mortality rate and complicated with RDW and hemoglobin groups**. Hb, hemoglobin; RDW, red cell width distribution.

In order to rule out the possibility that RDW effect on severe morbidity and mortality was unrelated to anemia, we compared complicated hospitalization in patients with hemoglobin (Hb) levels less than 110 g/L and higher levels of Hb. The two groups were examined in patients with normal and elevated RDW. As depicted in Figure 2, patients with normal RDW had no difference in severe morbidity or mortality regardless of Hb levels. On the other hand, elevated RDW was associated with a significant increase in both complicated hospitalization and 90-day mortality rates irrespective of Hb levels.

## Discussion

This study shows that in young patients with CAP, elevated RDW levels on admission either alone or in combination with abnormal levels of WBC are associated with significant higher rates of mortality and complicated hospitalization. RDW level as a prognostic marker was unrelated to hemoglobin levels on admission.

RDW is a simple laboratory test used to evaluate variance in the size and form of red blood cells. It is an important marker in the differential diagnosis of anemia. However, RDW was shown to be a predictor of severe morbidity and mortality in several cardiac problems [10-12,14]. There is a large body of evidence showing that RDW is a strong predictor for an adverse outcome in these conditions, equivalent to other known markers, such as low ejection fraction, high New York Heart Association Functional Classification (NYHA) score and elevated serum B-Type Natriuretic peptide (BNP) levels [15]. Moreover, it was recently reported that RDW is a strong predictor of mortality in several other conditions such as obesity, malignancies, and chronic kidney disease [16]. Concordant with our findings, Wang et al. reported recently that a graded independent relation between higher RDW and adverse outcomes in ICU patients [17].

Several explanations were suggested to this surprising finding. Abnormal RDW usually suggests one or more chronic condition that accompanies significant heart failure, such as anemia or nutritional deficit. Another mechanism that was suggested is the release of cytokines in response to inflammatory stress. These cytokines block the activity of erythropoietin and cause production of ineffective red blood cell and elevated RDW [18,19]. Lippi et al found a correlation between high RDW and elevated indexes of inflammation, such as elevated erythrocyte sedimentation rate (ESR) and C-reactive protein (CRP). This correlation was independent of concomitant diseases, and was demonstrated even when anemic patients were excluded from the statistical analysis [20].

Our data demonstrated that RDW is a valuable and sensitive marker for a high level of inflammatory activity in young patients with CAP, and it is independent of hemoglobin levels. The mechanism underlying the association between high levels of RDW and adverse outcome in patients hospitalized with pneumonia is unknown. RDW is elevated in conditions in which there is ineffective red cell production or increased destruction of red blood cell. RDW may indicate a severe inflammatory process, with impact on bone marrow function and iron metabolism. Another option is that RDW is an earlier marker of prognosis, and it may appear earlier in the complex process of anemia than low levels of hemoglobin. As noted before, high RDW levels may represent a culmination of multiple pathophysiologic processes occurring in acute inflammatory and infectious state.

Measuring RDW on admission is relatively simple and can be done during routine evaluation at the emergency room. Our data suggest that it may offer the physician another tool in the process of decision making whether to admit a patient diagnosed with CAP, in addition to the various scores that were developed in recent years for this purpose. Indeed, if RDW is proven to be a prognostic factor in older patients admitted with CAP, it might even be incorporated into such scores. In concordance with our data, several studies demonstrated the relation between mortality and elevated RDW in middle aged and older adults [21,22]. To our knowledge, our study is the first to show the poor prognostic effect of elevated RDW in young patients with CAP.

The major limitation of this study is its retrospective nature. In addition, our study examined RDW as a prognostic factor only in patients 60 years old and less. The majority of mortality and severe mortality in patients with CAP is in elderly patients, many of them have chronic hematologic and inflammatory co-morbidities. A larger cohort of this complex population is needed to clarify RDW as an independent risk factor in this population and to validate it prospectively including other inflammatory markers such as CRP, procalcitonin and IL-6. Notably, our study was planned to detect the association between elevated RDW and prognosis of CAP; however, we did not examine whether this observation is unique to CAP or could be demonstrated in other inflammatory and infectious states.

## Conclusions

In young patients with CAP, elevated RDW levels are associated with significant higher rates of mortality and severe morbidity. RDW as a prognostic marker was unrelated with hemoglobin levels on admission.

## Key messages

• Elevated RDW on admission is a recently described risk factor for adverse outcome in cardiac problems.

• This study demonstrates that elevated RDW in young patients hospitalized with CAP is a significant risk factor for complicated hospitalization and in-hospital mortality.

• Conceivably, measuring RDW in the emergency department might offer the clinician a simple yet powerful tool in the process of decision making whether to admit patients with CAP.

## Abbreviations

AUC: area under curve; BNP: b-type natriuretic peptide; BUN: blood urea nitrogen; RDW: red cell distribution width; CAP: community acquired pneumonia; CI: confidence interval; CRP: c-reactive protein; ESR: erythrocyte sedemintation rate; NYHA: New York Heart Association Functional Classification; OR: odds ratio; WBC: white blood cell.

## Competing interests

The authors declare that they have no competing interests.

## Authors' contributions

EB was involved in the conception, defining the aims, designing the study, analysis of data, and writing and revising the final manuscript. ED was involved in designing the study, data acquisition and analysis, and writing and revising the manuscript. YK and YM assisted in data acquisition and manuscript revision. BFM assisted in data acquisition, manuscript revision, supplied some of the references concerning the RDW section and participated in revising the methods and results of this specific section. ZSA was involved in the conception, defining the aims, designing the study, analysis of data, preparing the figures and tables, writing, revising and submitting the manuscript and is the guarantor of the paper. All authors have read and approved the manuscript for publication.
